# Implementation and evaluation of a driving cessation intervention to improve community mobility and wellbeing outcomes for people living with dementia: study protocol of the ‘CarFreeMe’ for people with dementia program

**DOI:** 10.1186/s12877-019-1074-6

**Published:** 2019-03-04

**Authors:** Theresa Scott, Jacki Liddle, Geoffrey Mitchell, Elizabeth Beattie, Nancy Pachana

**Affiliations:** 1 School of Psychology, The University of Queensland, St Lucia, Queensland 4072 Australia; 2 School of Information Technology and Electrical Engineering, The University of Queensland, St Lucia, Queensland 4072 Australia; 3 Primary Care Clinical Unit, Faculty of Medicine, The University of Queensland, RB&W Hospital, Herston, Queensland 4006 Australia; 4 Dementia Centre for Research Collaboration, School of Nursing, Queensland University of Technology, Kelvin Grove, Queensland 4059 Australia; 5 School of Psychology, The University of Queensland, St Lucia, Queensland 4072 Australia

**Keywords:** Dementia, Driving, Driving cessation, Older adults, Intervention, Support, Lifespace, Mobility

## Abstract

**Background:**

Giving up driving is a pivotal life event and universal challenge for people living with dementia and their families, and a complex area of clinical practice for health professionals who monitor driving cessation. The best outcomes are for individuals to plan for and eventually cease driving, however with insufficient support programs in place, many avoid the issue until it is reaches a crisis point. This program of research investigates a comprehensive support- and education-based intervention targeted at people living with dementia and their care partners who are managing driving cessation. The primary aim of this research is to determine the effectiveness of the program through a cluster randomized controlled trial.

**Methods/design:**

The intervention (CarFreeMe) is an intensive program delivered by a trained health professional that addresses practical and emotional needs relevant to driving cessation. The seven module program is person-centred, covering awareness raising, adjustment, and practical support that is individualized according to geographic location and the particular goals and preferences of participants. A cluster randomized controlled trial will evaluate the effectiveness of the program. Evaluation will take place pre-intervention, immediately following, and three months post-intervention. Clusters are randomized to either intervention or usual treatment. Participants within clusters will be recruited via primary and secondary care clinics, community agencies, service providers, local media, social media, support groups, and word of mouth. The primary outcome measure for persons with dementia and their care partners is lifespace, collected via (i) smartphone GPS technology and (ii) self-reported number of episodes away from home (during the past week). Secondary outcomes include safe alternative transport status, wellbeing, depression, anxiety, and self-efficacy, which will be collected from dyads. Caregiving strain will be collected from care partner/family member only. A process evaluation of the intervention will also be undertaken.

**Discussion:**

There is an urgent need for therapeutic approaches to supporting people living with dementia and their families to negotiate the complex decision making involved in deciding to change their approach to driving. The driving cessation intervention may fill an important gap in service delivery to people living with dementia who are adjusting to life without driving.

**Trial registration:**

Australian and New Zealand Clinical Trials Registry ACTRN12618000388213, 15 March 2018.

## Background

Stopping driving impacts the health and quality of life of people with dementia and their care partners. It also poses considerable challenges to health professionals who monitor driving issues and to those involved in testing and licensing. Without intensive practical and emotional support to plan for, and eventually, cease driving, people with dementia are at high risk for increased depression, anxiety, grief, social isolation, unsafe and unlicensed driving and injury. We have developed a comprehensive support- and education-based intervention targeted at people with dementia and their care partners and family members to manage driving cessation. The intervention is a translation of a proven driving cessation intervention for people without cognitive decline and has been trialled in an intensive case study. This project investigates the effectiveness of the evidence-based driving cessation intervention for people with dementia and their family members who are retiring from driving, through a cluster randomized controlled trial.

A diagnosis of dementia does not mean the immediate cessation of driving [1, 2]. However, at some stage people with dementia will need to stop [3]. Without necessary support programs in place, many people living with dementia avoid the issue until it is reaches a crisis point [4]. Health professionals acknowledge the complexity of driving and driving cessation issues for people with dementia, and studies identify that their lack of knowledge and confidence in managing driving cessation issues may lead to reluctance to do so [5]. Some drivers with dementia avoid their responsibility to stop driving even when their licence is terminated for emotional, logistical, and mobility reasons, or due to a lack of insight and awareness of the dangers [2, 4, 6]. Driving cessation can diminish ‘lifespace’ – defined as the extent of movement through the environment that captures lived community mobility [7] – constricted lifespace may lead to more rapid cognitive decline [8–10].

Timing of the decision to stop driving is critically important. If the decision to cease driving is delayed, then loss of insight into declining driving abilities exacerbates the challenges of stopping [3]. The best outcome is for people with dementia and their care partners to plan early, and eventually cease driving. However, it is an extremely personal and difficult issue for people with dementia, and one that care partners and families fear confronting [11]. Furthermore, with limited available information about how to evaluate fitness-to-drive in primary care, and no practical interventions for people with dementia, it is a poorly resourced area of clinical dementia care. Despite the concerns for safety and recognition of the impact of driving cessation on quality of life, health and wellbeing of people with dementia and their care partners, there are currently no theory-driven, empirically tested interventions to facilitate driving cessation for people with dementia in routine clinical practice within Australia. The CarFreeMe (for people with dementia) intervention aims to fill the gap.

### Preliminary work

The researchers have developed a comprehensive support and education-based driving cessation intervention, which is a translation of an effective community-based psychosocial intervention for people *without* cognitive decline: ‘CarFreeMe’ (formerly known as UQDRIVE) [12]. The translated intervention is aimed at the unique needs of drivers with dementia and their families. The effectiveness of the original CarFreeMe program, aimed at improving outcomes of driving cessation for older people without cognitive decline, has been systematically explored with a general older adult population in a randomized controlled trial [13]. The dementia specific intervention was pilot tested with a sample of drivers with dementia who had stopped driving and their family members in Brisbane, Queensland Australia [unpublished thesis]. Improvement in community mobility outcomes, self-efficacy [13], individualized transport and lifestyle goals [14], and high levels of satisfaction [15] have been reported by participants within CarFreeMe programs. The current program of research will implement a cluster randomized controlled trial (cRCT) examining face-to-face delivery of the dementia specific intervention to people living with dementia in the community, in Queensland, Australia.

### Aims and objectives

The primary aim of this study is to assess the impact of participating in the CarFreeMe intervention on lifespace compared to usual care, for people with dementia. The secondary aim is to assess the impact of CarFreeMe on quality of life, wellbeing, and self-efficacy for people with dementia and their care partners, compared to usual care.

The objectives are to:

- Maintain or increase community mobility outcomes for people with dementia who are transitioning to driving cessation (or have recently ceased driving);
- Increase use of safe alternative modes of transport;
- Evaluate the impact of the intervention on self-efficacy, wellbeing, and community participation of people with dementia;
- Evaluate the impact of the intervention on self-efficacy, wellbeing, and community participation of the care partners/family members of people with dementia.

It is predicted that compared with usual care, the driving cessation intervention:

1. Maintains or increases *lifespace* – as measured by metrics collected via a custom Smartphone app;
2. Maintains or increases *episodes away from home* and using safe modes of transport – as measured by metrics collected via Smartphone and App, and self-report;
3. Results in participants:

- greater self-efficacy in dealing with driving cessation decision making;
- higher measures of wellbeing;
- decreased measures of depression, anxiety, isolation, and stress.

## Methods

### Design

**Fig. 1:**
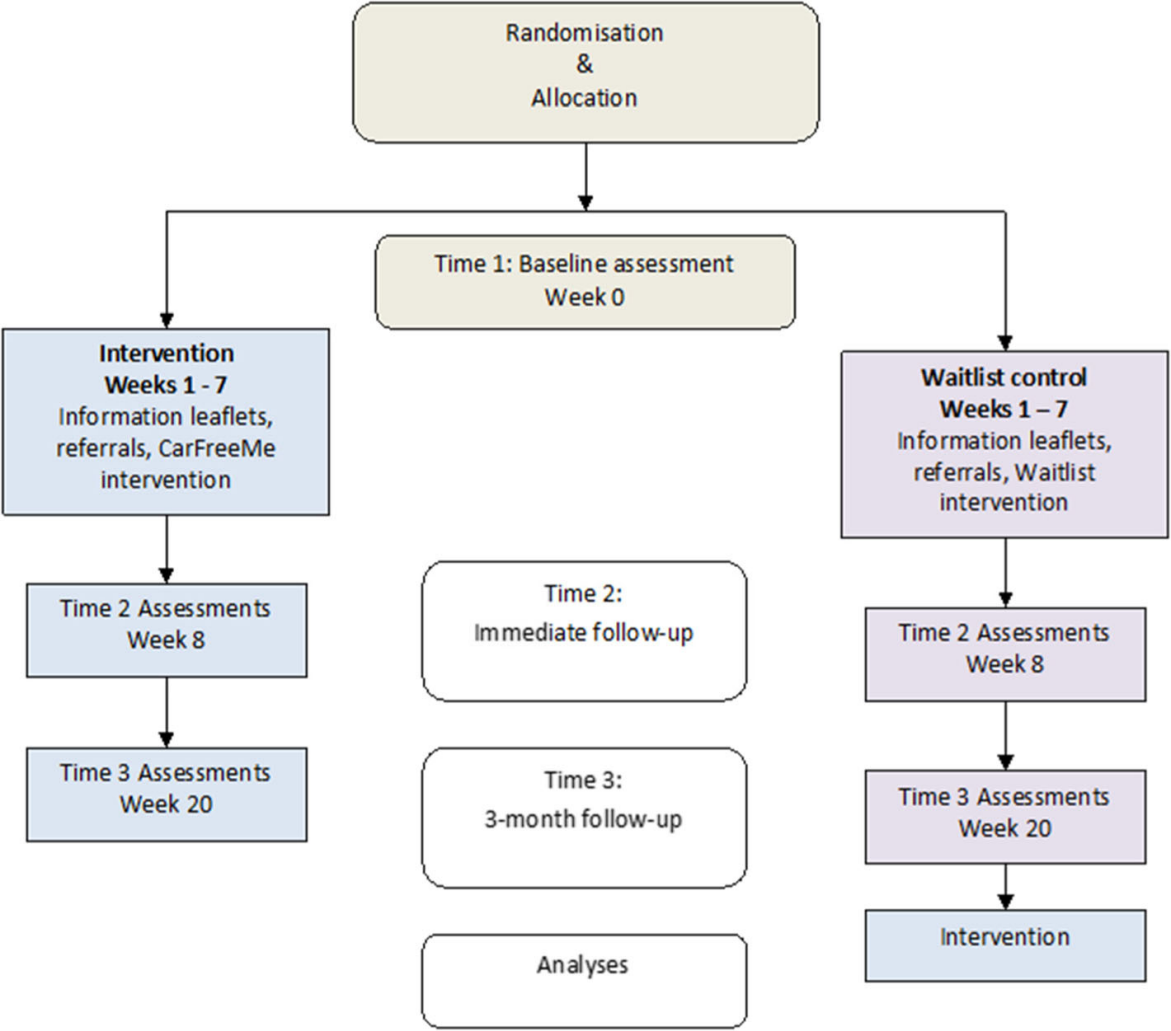
Abridged CONSORT diagram

A cluster randomized controlled trial will be conducted with regions (electoral districts) used as the unit of randomisation, to minimize the risk of contamination between conditions. Cluster randomisation aims to also address possible attrition, as participants will not be made aware of an alternative group. Clusters will be matched so that pairs of regions are as similar as possible on demographics, and potential local area transport and health infrastructure, to increase efficiency [16]. Clusters will be randomized using concealed allocation to either the intervention or waitlist control group. Randomisation to treatment or wait-list control condition will occur before baseline measurement. During a period of baseline data collection, no cluster will be exposed to the intervention. After baseline, one will proceed to the intervention arm, while the other will proceed to the wait-list ‘treatment as usual’ (ie. no intervention, written information, pamphlets) control arm – to receive the intervention at a later time (after Time 3, see Fig. 1).

### Study setting

The study will be set in the general community in Queensland and Northern New South Wales, Australia.

### Participants

#### Eligibility criteria

*Persons with dementia* will be older people (aged 65 years and over), male or female, with mild cognitive impairment or mild or moderate dementia of any type, and driving or driving cessation is a current issue for them. Persons living with dementia will be able to provide informed consent to participate or (where determined necessary) to have a care partner that can provide proxy informed consent on their behalf. People with a diagnosis of younger-onset of dementia are excluded from this study because the program has not been translated for people with younger-onset dementia as yet. Non-English speaking participants will be excluded from participating because the intervention at present does not cater to a non-English speaking audience. Potential participants with dementia will be screened using the Clinical Dementia Rating scale (CDR) [22]. Participants with scores of 2 or less will be eligible to participate (0.5 = very mild dementia, 1 = mild dementia, 2 = moderate dementia).

*Care partners/support persons* self-identified informal primary care partners of the retired/retiring driver with dementia are eligible to participate alongside that person. To be eligible support persons may be male or female, and must be aged 18 years and over, have the ability to communicate in English, and be providing ongoing support to the person with the dementia.

### Intervention description

CarFreeMe-for People with Dementia was developed in accordance with the four stages of the Medical Research Council’s framework (2000) for investigating health-related complex interventions. Stage 1 has been conducted in the preliminary work undertaken to identify the issues for driving cessation for people with dementia and their care partners. This informed the development of the seven-module CarFreeMe for People with Dementia intervention (see Table 1). The content, language, format and activities were adapted from a proven driving cessation intervention for people without cognitive decline [13] for people with dementia and additional modules created for family members – whose involvement is key. The adaptation was reviewed by an expert reference group and preliminary evaluations conducted within an intensive case study model. The CarFreeMe for People with Dementia intervention is designed to be person-centred and flexible in content and delivery, e.g. in-home, one-to-one, dyads, or small group settings. As part of its design, the intervention is individualized according to geographic location and the needs of participants. For example, older adults are encouraged to consider future transport arrangements, plan for lifestyle changes, form realistic expectations of life changes after driving cessation, and practice using alternative transport. Importantly, they are supported in their emotional adjustment to the role loss. The individualized program involves a combination of one-to-one sessions, groups, practical outings and activities with a local experienced health professional, as well as some home-based independent activities.

**Table 1:** Structure and content of the intervention for people living with dementia

Modules	Title and contact examples
Module 1	Living with dementia: focuses on the changes that may occur with dementia, and strategies to live positively.
Module 2	Balancing independence and safety: provides information about driving safety in later life and things for consideration regarding retirement from driving.
Module 3	Adjusting to losses and changes: covers changes that may occur to lifestyle and feelings of loss and grief that may result from retiring from driving. It also includes strategies to use to help with adjusting.
Module 4	Experience of retiring from driving: covers what it can be like to give up driving. Stories from other retired drivers and family members are included to highlight different ways that people have adjusted to giving up driving.
Module 5	Alternative transport: covers the range of alternatives to driving that may be useful and ideas of where to find out more.
Module 6	Lifestyle planning: covers things to consider in planning for achieving a balanced lifestyle.
Module 7	Advocacy and support: focuses on the services that are available to participants and the steps to take to improve the service/s, and to make service providers aware of these needs.

The intervention includes seven modules that cover education and practical support, delivered by a health professional who is trained in, for example, occupational therapy, psychology, social work or nursing, and who is experienced in working in community care with older adults living with dementia and their family members. It is anticipated that trained health professionals will deliver the intervention to people with dementia and their families in their homes and/or community settings, in individual, or group sessions, or a combination of both – depending upon participant preference.

Data collection will be conducted face-to-face with the person with dementia and their care partner by a research assistant who is blinded to the treatment condition that participants are randomized to.

### Control condition/comparator

Participants assigned to the control condition will receive ‘as usual’ treatment, which will include provision of information leaflets, for example information regarding driving and dementia and contact details of service providers.

### Recruitment and implementation plan

#### Participant timeline

The intervention includes seven modules that take approximately 60–90 min to deliver, and at least one practical outing. Three temporal measurement phases are involved in the trial. These include week one baseline assessment (Time 1), week 8 first follow-up post-intervention (Time 2), and three-month follow up (Time 3) approximately week 20, as shown in Fig. 1. Clusters will be randomized to the sequence of treatment (i) Baseline > Intervention > Follow up, or (ii) Baseline > Control > Follow up > Intervention), as shown in Fig. 1. That is, participants in the wait-list control group will be offered the driving cessation intervention after the three-month follow up assessment.

#### Sample size

The sample size calculation for this study used a power analysis applying the mean, and standard deviation (SD), and the minimum detectable difference between populations means = 1, for episodes away from home, obtained from a background study [13]. Allowing for 20% dropout and assuming detectable difference = 1, *SD* = 3.16 and ICC = 0.05. For 80% power with alpha set at 0.05, and the minimum detectable difference between population means, then with an average of 15 dyads per cluster, the minimum number of clusters required is 10. However, more clusters will be included to reach a minimum sample size.

#### Recruitment

Recruitment will begin at the cluster level. Potential participants – dyads for whom driving cessation is a current issue (including those still planning, those who have stopped and those who should have stopped) – will be recruited on a rolling basis across 2.5 years via primary and secondary care clinics, community agencies, service providers, local media, social media, dementia support groups, and word of mouth. Participants will be invited to contact the researchers to obtain further details of the study, or to give their verbal permission to their health professional (e.g. general practitioner, neuropsychologist,) to pass on their contact details for the researchers to follow up, for the purpose of providing more information about the study.

#### Outcomes and assessment tools

##### Main outcome measure

The main outcome measure for persons with dementia and their family members is lifespace – the geographic space in which a person lives and conducts their activities. It is recognized that having dementia is likely to constrict a person’s lifespace and reduce their community participation. Lifespace will be captured through two measures, (i) the number of episodes away from home (during the past week); and (ii) novel Smartphone GPS technology used to passively measure lifespace metrics for people with dementia and their care partners. Participants will carry a study smartphone in a comfortable case (provided to them by the researcher – on loan and at no cost) for the period of one week pre- and one week post-intervention and one-week at three-month follow-up. Support is provided to make sure the purpose of the phone is understood and remembered by participants. Data stream to a secure portal and can enable the research team to identify if a problem with data collection has occurred. Family members are asked to remind the person with dementia to wear the smartphone. While the limitations of this method are acknowledged, strategies will be in place to mitigate these. This type of data collection approach has been used successfully with this population. An ‘episode away from home’ is defined by a single occurrence of leaving the home, counted for the previous week, which is collected by Smartphone and App from persons with dementia, and by self-report from them and/or their care partners.

##### Secondary outcome measures

‘Safe transport status’ is the major secondary outcome measure. We will collect information from participants about the mode of transport used for each episode away from home (e.g. walk, taxi, bus), counted for the previous week, and about any concerns they had about the mode of transport used (e.g. safety). Other secondary outcome measures include wellbeing, depression, anxiety, self-efficacy and self-performance and isolation, which will be collected from dyads; as well as caregiving strain collected from carer/family member only (see Table 2). At baseline, general questions related to demographics, mobility, proximity to services, usual transport used, community involvement, socioeconomic status, and the circumstances surrounding driving cessation (e.g. participant is the only driver in household, participant’s readiness to transition to retired driver), will be collected.

**Table 2:** Pre- and post-intervention measurement tools relating to persons with dementia and family members/care partners, collected at Times 1, 2, and 3

Person with dementia	Family Member	Measure
Lifespace; Episodes away from home	Lifespace; Episodes away from home	Lifespace metrics via Smartphone GPS data; Self-report, Life Space Questionnaire [19]
Mode/s of transport used in previous week	Mode/s of transport used in previous week	Semi-structured Interview
Readiness for transitioning to non-driver		Assessment of Readiness for Mobility Transition [20]
Social Isolation	Social Isolation	Loneliness Scale [21]
Wellbeing/Quality of life	Wellbeing/Quality of life	Personal Wellbeing Index [22]
	Caregiver Strain	Caregiver Burden Index [23]
Self-efficacy - Transport and lifestyle	Caregivers Self-efficacy	Transport and Lifestyle Self Efficacy [13]
Depression and Anxiety symptoms	Depression and Anxiety symptoms	Geriatric Depression Scale [24]; Geriatric Anxiety Inventory [25]
Individualized goal setting (and achievement of goals)	(completed together)	Canadian Occupational Performance Measure [26]
Perceptions of needs and experiences, feedback	Perceptions of needs and experiences, feedback	Semi-structured interviews with sub-sample

##### Interviews

In addition, we aim to interview a sub-sample of participants – participants in the intervention condition – at (i) pre- and (ii) post-intervention to ascertain an understanding of their experiences, decision-making, timing of driving decisions and events that influenced these decisions, and about their experiences of the intervention (e.g. what worked well, didn’t work well, what made a difference, whether their needs were met, and what issues they experienced going through the process). These interviews will be conducted by a researcher by telephone or in-person and will therefore be separate from blinded assessments at Times 2, and 3. The interviews will be approximately 15–30 min duration. These will be audio recorded and transcribed verbatim and later thematically analysed. Those that consent to being followed up by telephone or email will be given an opportunity to member check the researchers’ findings in relation to themes that emerge from the transcribed data. These participants will therefore be contacted again, after all data are collected and analysed.

##### Determining severity of cognitive decline

The CDR scale will be used to determine participants’ severity of dementia, for the purposes of sample description. A global CDR of 0 indicates no dementia. A CDR of 0.5 represents very mild dementia or in some cases with minimal impairment, uncertain or questionable dementia. A CDR of 1, 2, or 3 corresponds to mild, moderate, or severe dementia, respectively. Participants’ score on the CDR [17] screening tool will be used to aggregately describe the sample in any written reports or manuscripts (e.g. X participants with mild dementia, X with moderate dementia, according to the CDR).

### Process evaluation

A process evaluation will explore the implementation, receipt, and setting of the intervention and help in the interpretation of the outcome results. In trials, where the “same” intervention may be implemented and received in different ways, process evaluations can provide greater explanatory power and understanding of the generalisability of the intervention [18].

### Treatment fidelity

Blinded assessors will be trained to deliver the standardized assessment measures and materials. Health professionals will be registered and will be trained in delivery of the intervention via online training and written materials. Further, health professionals will be supported, where needed, by master trainers while delivering the driving cessation intervention. To monitor fidelity of delivery, participants’ sessions will be detailed in an Excel file by health professionals, e.g. documenting date, topic of session, location, activities, homework, etc. These files will be reviewed by research staff.

### Randomisation and blinding

The research will adopt a single blind design. Further blinding is not feasible given the nature of the intervention. The researcher will generate the random allocation sequence (using simple randomization methods, e.g. sealed envelopes) before baseline assessment is conducted. Clusters, which are matched by the researcher, based on available infrastructure in health and transport in the region, are randomized using concealed allocation to either the intervention or waitlist control group. Clusters will be matched so that pairs of regions are as similar as possible on demographics, population density, and transport and infrastructure, to increase efficiency [16]. Participants will be allocated to either intervention or control condition, based on the area (cluster) that they live in. However, assessors will be blinded to the group assignment of individual participants and will not know which condition the clusters are allocated to. We will ask participants not to inform the independent assessors, if they themselves know, to the best of their ability.

### Procedure

A researcher will have initial telephone contact with potential participants – ie. older adults who self-select for the study. The researcher will answer any questions that potential participants may have and provide further information, and conduct the screening process. A researcher will ask questions of the caller about (as appropriate) their own or the retired/retiring driver’s age, the types of memory problems he or she is having, and their driving situation, to determine their eligibility. Participants with dementia or their family member/support person will be asked to report the individual’s diagnosis of dementia and stage of dementia, ie. participants in the mild to moderate stage dementia will be screened through self-report or proxy-report and the CDR, and will be eligible to participate. If appropriate the researcher will make an appointment for an assessor to attend the participants’ home to conduct baseline assessment. Participants will be given the option to re-consider their participation after their initial contact and before the baseline assessment, or at any time during the study. A follow-up telephone call will be made to potential participants prior to the assessment commencing, by way of reminder, to answer any further questions and confirm their participation.

Initial assessment will take place after obtaining written informed consent. Based on cluster randomisation, participants will be assigned to either the immediate intervention group, or to the wait-list control group. An assessor, who is blind to group allocations will attend in person (e.g. participants’ homes) to conduct all assessments. Following initial assessments, participants will be given a choice of home visit, telephone, or videoconference, for the next two data collection time points (ie. Times 2 and 3, as shown in Fig. 1). The intervention group will receive the intervention approximately two weeks after baseline assessment, and the control group will be offered to receive the intervention after Time 3 assessment. The wait-list control group will receive regular mailed brochures (ie. treatment as usual) and telephone calls throughout the wait period.

### Consent and withdrawal procedures

As outlined above, written informed consent will be obtained from participants at the first meeting, immediately prior to participants’ first assessment. That is, after participants are briefed about the study once again, they will be given the opportunity to ask questions, consider their participation and if they agree, to indicate their agreement by signing the consent form and then proceeding with baseline assessment. Participants’ continued consent and assent will be checked throughout the study. Participants who withdraw from the study will be given the opportunity to also withdraw their data to that point if they wish to. We will collect information about the reasons why participants withdraw from the study.

### Data analysis

Baseline demographic and cluster characteristics will be compared between the intervention and control groups. Data will be analysed using random effects models with maximum likelihood estimation. All analyses will be conducted at the individual and aggregated cluster levels using generalized estimating equations to adjust for clustering effects within geographical location, and adjusting for intracluster correlations between individuals, and according to intention to treat principles.

Hypotheses 1 & 2. Increase in lifespace and the rate of episodes away from home will be analysed at the cluster level using a linear mixed effects model with random intercepts used to analyse the effect of the intervention on number of episodes away from home.

Hypotheses 3 & 4. Testing the effect of the intervention on self-efficacy, wellbeing, depression symptoms, anxiety symptoms, and social isolation, will involve linear mixed effects model.

Qualitative analyses will include thematic analysis of the semi-structured interviews with participants. Coding will be conducted and verified by minimum three members of the research team. The group will convene to review themes until consensus is reached.

### Data management

Data will be collected electronically via tablets made available to blinded assessors, or in hard copy. Data will be stored on a computer hard-drive that is protected by password. Any data that are collected in hard copy will be stored in a locked cabinet at the Principal Researcher’s office at The University of Queensland. Any written results or reports relating to the study will be de-identified. The results will be disseminated to stakeholders in brief reports, presentations, and academic publications.

## Discussion

Giving up driving is a pivotal life event and universal challenge for people living with dementia and their families, and the health professionals who monitor driving with their patients with dementia. The best outcomes are for individuals to plan for and eventually cease driving, however with insufficient support programs in place, many avoid the issue until it is reaches a crises point. With increasing numbers of older adults driving in later life and increasing numbers of people living with dementia, there is an urgent need to upskill health professionals to manage the complex issues around dementia and driving cessation, and to explore the most cost-effective and timely ways to deliver support to people living with dementia. The driving cessation intervention – aimed at enhancing participants’ community mobility and wellbeing – may fill an important gap in service delivery to people living with dementia who are adjusting to life without driving.

Future research aims are to translate the driving cessation intervention for people with dementia into routine clinical practice. The process evaluation will contribute valuable information to understanding optimal processes, for example timing and number of sessions, in determining a feasible translation strategy.

## Abbreviations

APP: (software) Application
CDR: Clinical Dementia Rating
cRCT: Cluster randomized controlled trial
GPS: Global positioning sequence
NHMRC: National Health and Medical Research Council
OT: Occupational Therapist
